# Measuring self-regulation in everyday life: Reliability and validity of smartphone-based experiments in alcohol use disorder

**DOI:** 10.3758/s13428-022-02019-8

**Published:** 2022-12-12

**Authors:** Hilmar Zech, Maria Waltmann, Ying Lee, Markus Reichert, Rachel L. Bedder, Robb B. Rutledge, Friederike Deeken, Julia Wenzel, Friederike Wedemeyer, Alvaro Aguilera, Acelya Aslan, Patrick Bach, Nadja S. Bahr, Claudia Ebrahimi, Pascale C. Fischbach, Marvin Ganz, Maria Garbusow, Charlotte M. Großkopf, Marie Heigert, Angela Hentschel, Matthew Belanger, Damian Karl, Patricia Pelz, Mathieu Pinger, Carlotta Riemerschmid, Annika Rosenthal, Johannes Steffen, Jens Strehle, Franziska Weiss, Gesine Wieder, Alfred Wieland, Judith Zaiser, Sina Zimmermann, Shuyan Liu, Thomas Goschke, Henrik Walter, Heike Tost, Bernd Lenz, Jamila Andoh, Ulrich Ebner-Priemer, Michael A. Rapp, Andreas Heinz, Ray Dolan, Michael N. Smolka, Lorenz Deserno, the ReCoDe-Consortium

**Affiliations:** 1 Department of Psychiatry, Technische Universität Dresden, Dresden, Germany; 2 Department of Child and Adolescent Psychiatry, Psychosomatics and Psychotherapy, Centre of Mental Health, University of Würzburg, Margarete-Höppel-Platz 1, 97080 Würzburg, Germany; 3 Max Planck Institute for Human Cognitive and Brain Sciences, Stephanstraße 1, 04103 Leipzig, Germany; 4 Max Planck University College London Centre for Computational Psychiatry and Ageing Research, London, UK; 5 Wellcome Centre for Neuroimaging (WCHN), University College London, London, UK; 6 Department of eHealth and Sports Analytics, Faculty of Sport Science, Ruhr-Universität Bochum (RUB), Bochum, Germany; 7 Mental mHealth Lab, Institute of Sports and Sports Science, Karlsruhe Institute of Technology (KIT), Karlsruhe, Germany; 8 Department of Psychiatry and Psychotherapy, Central Institute of Mental Health, Medical Faculty Mannheim, Heidelberg University, Mannheim, Germany; 9 Neuroscience Institute & Department of Psychology, Princeton University, Princeton, NJ USA; 10 Department of Psychology, Yale University, New Haven, CT USA; 11 Social and Preventive Medicine, Department of Sports and Health Sciences, Intra-faculty unit “Cognitive Sciences”, Faculty of Human Science, and Faculty of Health Sciences Brandenburg, Research Area Services Research and e-Health, University of Potsdam, Potsdam, Germany; 12 Department of Psychiatry and Neurosciences | CCM, Charité – Universitätsmedizin Berlin, corporate member of Freie Universität Berlin and Humboldt-Universität zu Berlin, Department of Pediatric Surgery, Augustenburger Platz 1, 13353 Berlin, Germany; 13 Center for Information Services and High Performance Computing (ZIH), Technische Universität Dresden, Dresden, Germany; 14 Department of Addictive Behavior and Addiction Medicine, Central Institute of Mental Health, Medical Faculty Mannheim, Heidelberg University, Mannheim, Germany; 15 Department of Clinical Psychology, Central Institute of Mental Health, Medical Faculty Mannheim, University of Heidelberg, Mannheim, Germany; 16 Department of Psychology, Technische Universität Dresden, Dresden, Germany; 17 State Key Laboratory of Cognitive Neuroscience and Learning, IDG/McGovern Institute for Brain Research, Beijing Normal University, Beijing, China; 18 BIH Visiting Professor, Stiftung Charité, Department of Psychiatry and Psychotherapy, Charité – Universitätsmedizin, Berlin, Germany

**Keywords:** Behavioral tasks, Smartphone, Reliability, Validity, Working memory, Stop signal task, Information sampling, Risk-taking

## Abstract

Self-regulation, the ability to guide behavior according to one’s goals, plays an integral role in understanding loss of control over unwanted behaviors, for example in alcohol use disorder (AUD). Yet, experimental tasks that measure processes underlying self-regulation are not easy to deploy in contexts where such behaviors usually occur, namely outside the laboratory, and in clinical populations such as people with AUD. Moreover, lab-based tasks have been criticized for poor test–retest reliability and lack of construct validity. Smartphones can be used to deploy tasks in the field, but often require shorter versions of tasks, which may further decrease reliability. Here, we show that combining smartphone-based tasks with joint hierarchical modeling of longitudinal data can overcome at least some of these shortcomings. We test four short smartphone-based tasks outside the laboratory in a large sample (*N* = 488) of participants with AUD. Although task measures indeed have low reliability when data are analyzed traditionally by modeling each session separately, joint modeling of longitudinal data increases reliability to good and oftentimes excellent levels. We next test the measures’ construct validity and show that extracted latent factors are indeed in line with theoretical accounts of cognitive control and decision-making. Finally, we demonstrate that a resulting cognitive control factor relates to a real-life measure of drinking behavior and yields stronger correlations than single measures based on traditional analyses. Our findings demonstrate how short, smartphone-based task measures, when analyzed with joint hierarchical modeling and latent factor analysis, can overcome frequently reported shortcomings of experimental tasks.

**Supplementary Information:**

The online version contains supplementary material available at 10.3758/s13428-022-02019-8.

## Introduction

Self-regulation, the ability to guide feelings and behaviors according to one’s needs and goals, relates to a range of outcomes, including somatic and mental health (Goschke, 2014; Moffit et al., 2011). Consequently, there is a growing interest in self-regulation across research domains, using a variety of measurement methods (Eisenberg et al., 2019; Nigg, 2017). Whereas questionnaires primarily capture explicit aspects of self-regulation, experimental tasks are designed to capture distinct cognitive and motivational mechanisms underlying self-regulation. Further added value of experimental tasks is their ability to manipulate physiological and brain states in a controlled manner. Research using experimental tasks thus promises an improved mechanistic understanding of phenomena such as self-regulation and its failures, and ultimately the development of targeted, mechanism-based treatments for psychiatric conditions such as substance use disorder (SUD).

However, although bespoke experimental tasks can outperform questionnaires in measuring distinct processes, linkage to real-life outcomes has so far been less successful than questionnaire-based measures. In a recent study, Eisenberg et al. (2019) assessed the ecological validity of 22 questionnaires and 37 task measures. While questionnaires modestly predicted real-world outcomes, experimental tasks showed no relationship to real-world outcomes. In the domain of SUD, Ekhtiari et al. (2017) reviewed studies that attempted to link decision-making tasks to drug use and concluded that these measures may not be sufficient to predict drug use in real life. These findings mirror a more widely held view that experimental tasks lack “realism” and generalizability (Falk and Heckman, 2009).

Here, we argue that experimental tasks do not inherently lack real-world relevance, but instead that shortcomings regarding their psychometric properties can explain their lack of realism. An important psychometric property is test–retest reliability—or a task’s consistency in measuring between-participant differences. Recent reports revealed low test–retest reliability for many tasks (Enkavi et al., 2019; Hedge et al., 2018). Low test–retest reliability is a challenge when we relate one measurement to another (e.g., relating working memory to alcohol consumption in SUD)—because mathematically low reliability limits the observable correlation between two measures (Spearman, 1904/2010: Eq. 1).

1 $${r}_{observed}={r}_{true}\ast \sqrt{reliabilit{y}_x\ast reliabilit{y}_y}$$

As an illustration, if working memory and drinking have a high *true* correlation of .8, but working memory is measured with a low reliability of .31 (which is the median reliability of experimental tasks reported by Hedge et al., 2018), then any observable correlation between the two measures would mathematically decrease to an upper limit of .44. Thus, weak or no relations between task measures and real-life outcomes may simply arise from the low reliability of task measures (Enkavi et al., 2019; Hedge et al., 2018).

In addition to these psychometric shortcomings, experimental tasks—unlike questionnaires—are more difficult to deploy outside the laboratory in real-life scenarios (Zech et al., 2022). Most experimental tasks have been designed to run on laboratory computers, requiring specialized software and, in some cases, specialized hardware. This makes it difficult to link task measures to critical real-life events. For example, binge drinking happens occasionally and may take place in highly specific real-life environments (e.g., in the bar). Indeed, studies that successfully connected task measures to self-control failures used smartphones to measure self-control failures in the field (Berkman et al. 2011; Krönke et al., 2018, 2020a, 2020b, 2021a, 2021b; Lopez et al. 2014; Overmeyer et al., 2021). Some of these studies even suggest that state-like mobilization of control rather than trait self-control can explain self-control failures in daily life (Krönke et al., 2018). The lack of mobility of laboratory tasks makes it difficult to measure such state-like processes. Their lack of mobility also makes it difficult to target large samples of specifically vulnerable patient populations. Together with their low reliability, the lack of mobility of tasks could therefore explain the difficulty of linking task measures to real-life variables.

Recently, several lab-based tasks have been translated to smartphones, which makes it easier to deploy tasks in the field and in relevant populations (for a summary of advantages of using smartphones in behavioral studies, see Miller 2012 and Zech et al., 2022). However, especially when using smartphone tasks in longitudinal designs, they often need to be shorter than lab-based tasks to assure participant compliance outside the laboratory and in repeated testing sessions. Making tasks shorter (i.e., reducing the number of trials), however, reduces task reliability and thus further aggravates existing psychometric problems of experimental tasks (Miller & Ulrich, 2013; Rouder & Haaf, 2019; Smittenaar et al., 2015; Zech et al., 2022). Researchers consequently find themselves in a dilemma: Either they have to rely on long tasks that may sacrifice compliance even in the lab, or they use short tasks that may be unreliable.

**Fig. 1:**
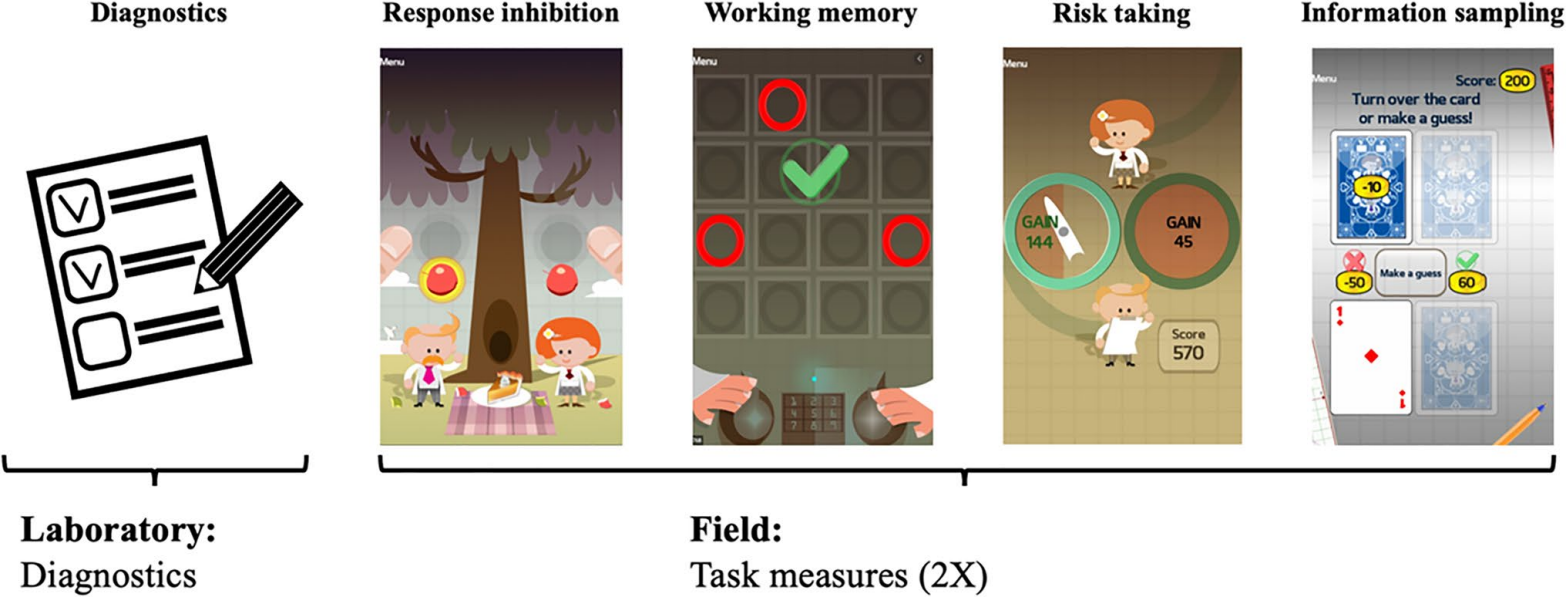
Illustration of study timeline and smartphone-based tasks. After an in-lab session for study inclusion and app installation, participants performed four tasks (twice in random order) from a customized version of the Great Brain Experiment (GBE, translated to German) at home. This included a response inhibition, a working memory, a risk-taking, and an information sampling task

Here, we thus outline an approach to overcome shortcomings in the applicability of experimental tasks to real-life scenarios: firstly, this entails moving task measures to platforms such as smartphones, which allows for the collection of rich longitudinal data in real-life contexts; secondly, it analytically exploits this longitudinal data to produce more reliable task metrics. Using this method, we test the reliability and construct validity of four smartphone-based tasks in a large (*N* = 488) sample of participants with mild to moderate alcohol use disorder. Little is known about the psychometric properties of experimental tasks in this population, although it is often the target of task-based research that strives toward clinical application (e.g., Heinz et al., 2020; Stavro et al., 2013; but see Kräplin et al., 2016). The tasks were gamified to increase participant engagement and designed to capture four distinct cognitive and motivational processes: working memory (McNab et al., 2015), response inhibition (Smittenaar et al., 2015), risk-taking (Rutledge et al., 2014), and information sampling (Hunt et al., 2016; see Fig. 1).

Beyond reliability, which is agnostic to what is measured, a theoretical assumption that tasks are particularly well suited to assess distinct cognitive-motivational processes requires the empirical investigation of their construct validity. In vulnerable populations, such as individuals with SUD, widespread cognitive alterations are well known (Hildebrandt et al., 2021) and may impede the detection of specific cognitive-motivational processes. Instead, multiple experiments may return a more general impairment, an undesirable scenario for mechanism-based research that aims to improve clinical stratification and ultimately individualized treatments. In fact, both reliability and construct validity do not depend on the task alone but also on the characteristics of an investigated sample (Knekta et al., 2019). When striving for future clinical applicability, there is an urgent need to assess psychometric criteria in populations such as people with SUDs. We therefore also assessed the tasks’ validity by analyzing their latent factor structure and by correlating the resulting factor scores with real-life measures of drinking.

Together, this study aimed to show how smartphones can be used to overcome shortcomings of experimental tasks by using joint modeling of longitudinal data from outside the laboratory to produce reliable and valid task measures in a clinical population diagnosed with alcohol use disorder.

## Results

### Study design

Data were collected as part of a larger research consortium on substance use disorder (SUD) employing a smartphone-based longitudinal Ecological Momentary Assessment (EMA) of up to 1 year (Heinz et al., 2020, see Materials and methods). After study inclusion, individuals with alcohol use disorder and associated comorbidities (Materials and methods, Table S1) commenced a smartphone-based data collection. On the first day of data collection, they completed four experimental tasks twice in pseudo-random order (randomized within each session; see Fig. 1), which allowed us to assess reliability and construct validity in *N* = 488 individuals. Of these, 373 participants (76.4%) completed the four tasks again 3 weeks later, allowing us to assess test–retest reliability at a longer retest interval. The tasks were taken from the Great Brain Experiment (GBE) app (Brown et al., 2014, see Materials and methods). Tests of response inhibition, working memory, risk taking, and information sampling were included and presented as games. In addition to completing the tasks, participants also self-reported how many days in the past 3 months they had consumed alcohol as well as how much alcohol they consumed on a regular drinking day. The study protocol was pre-registered (https://osf.io/9ze2u/).

### Reliability

We initially assessed split-half reliability (or internal consistency) for each task, and each of the two sessions. This indicates consistency of a measure within a session by splitting trials into two halves and comparing scores across each half. Subsequently, we analyzed test–retest reliability, the temporal consistency across two distinct sessions, comparing two analytic approaches in this regard. In a commonly used approach (Enkavi et al., 2019; Hedge et al., 2018; Rouder & Haaf, 2019), we computed task scores for each session separately and then examined test–retest reliability based on the resulting scores. We refer to this as a *separate modeling* approach. A problem in this popular approach is that it does not consider the dependency of data within participants (the hierarchical longitudinal structure of the data) and, both theoretically and empirically, this has been shown to lead to exaggerated residual variance, which depresses reliability (Brown et al., 2020; Rouder & Haaf, 2019; Waltmann et al., 2022). Therefore, we next analyzed the data with a *joint modeling* approach by modeling data jointly from both sessions using hierarchical mixed models (for details, see Materials and methods and Supplementary Materials). These models account for a dependency of data within participants, thereby regularizing scores by moving session scores towards participant means, with further regularization by moving participant means towards the sample mean. Indeed, Waltmann et al. (2022) showed that this joint modeling approach yields more accurate session score estimates, leading to both higher and more accurate estimates of reliability than a *separate modeling* approach. Test–retest reliability was assessed using intra class coefficients (ICCs), which compare the variance of interest—the between-participant variance—with the total residual variance (including systematic within-session variance, e.g., repetition effects). We report ICC(1) (Liljequist et al., 2019) as a primary outcome because it can be computed for separate and joint modeling approaches (see Supplementary Materials).

### Split-half reliability

For the risk taking task, split-half reliabilities for the gain and loss gambles were adequate (according to interpretations by Nunnally and Bernstein, 1994; *r*_*sb*_ *gain session 1* = .84; *r*_*sb*_*_gain session 2* = .91; *r*_*sb*_ *loss session1* = .77; *r_sb_loss_session_2* = .82) but lower for mixed gambles (*r*_*sb*_*_mixed_session_1* = .67; *r_sb_mixed_session_2* = .71). For the information sampling task, split-half reliabilities were adequate (*r_sb_session_1* = .86; *r_sb_session_2* = .86). Split-half reliabilities for the working memory task and for the inhibition task could not be analyzed in this way due to their adaptive task design (see Methods section).

### Test–retest reliability

**Fig. 2:**
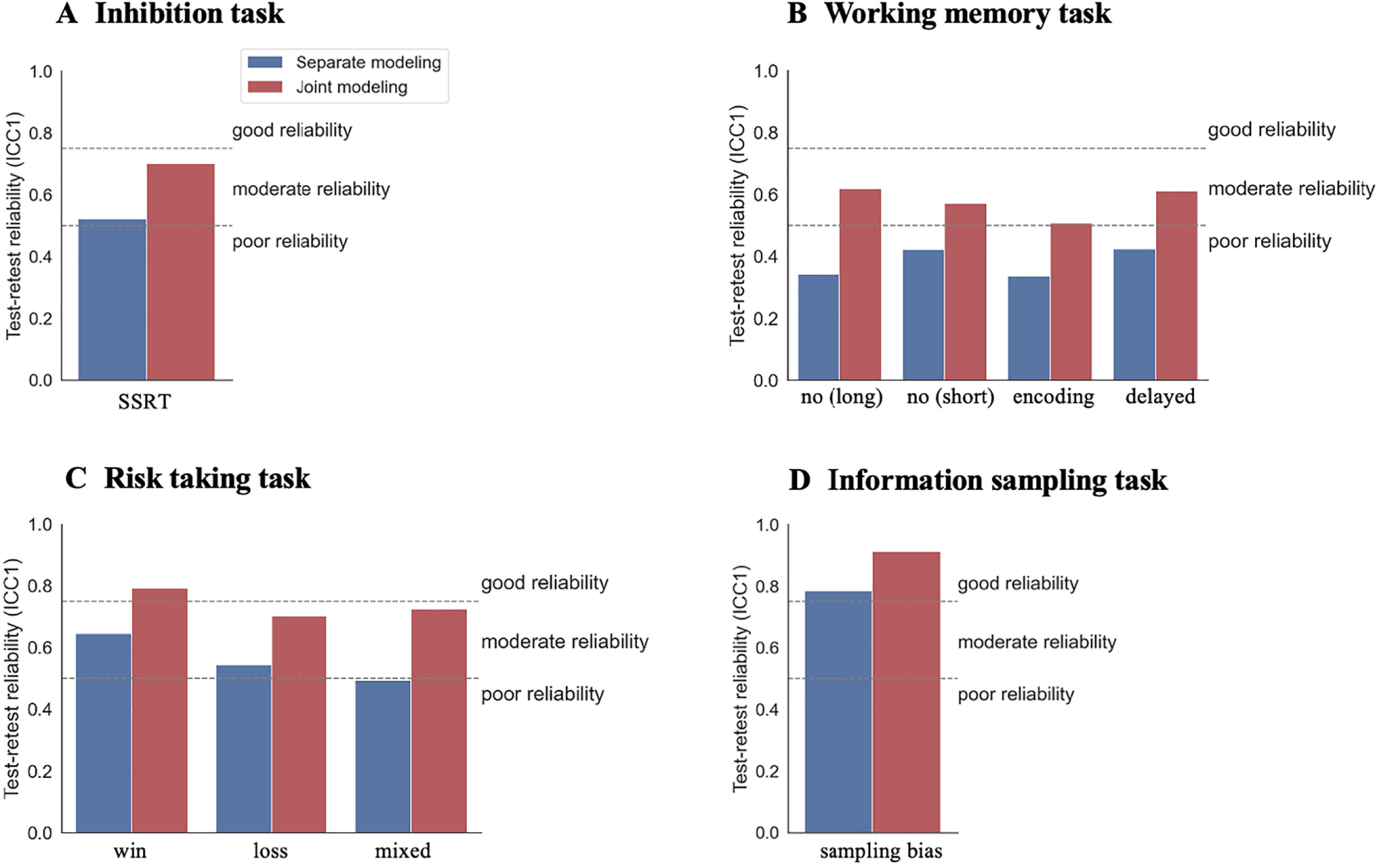
Test–retest reliabilities (ICC1s) for the four tasks split by separate (*blue*) vs. joint approach (*red*). Panels show reliabilities for the main outcomes of the experimental tasks. **A** Stop signal reaction times (SSRTs) for the inhibition task. **B** Four task outcomes of the working memory task, consisting of no distractor with long encoding time (no [long]), no distractor with short encoding time (no [short]), distractor cues presented at the same time as the patterns (encoding), and distractors presented after the patterns (delayed). **C** Three main outcomes of the risk-taking task, consisting of risk-taking in a gain context (win), risk-taking in a loss context (loss), and risk taking when gains and losses are mixed (mixed). **D** Main outcome of the information sampling task, the degree of sampling bias

Test–retest reliability increased for all tasks when calculating scores based on the joint compared to the separate modeling approach (see Table 1 and Fig. 2). The inhibition task had moderate reliability when scores were calculated based on separate modeling (ICC1 = .51; according to interpretations by Koo and Li, 2016), but good reliability when scores were calculated based on joint modeling (ICC1 = .70). For comparison^1^, the reliabilities of SST scores reported by Hedge et al. (2018) ranged between .36 and .49. The working memory task had poor reliability in all conditions when scores were calculated based on separate modeling (ICC1s ≤ .43), but this reliability increased to moderate levels when scores were calculated based on joint modeling (ICC1 ranging from .51 to .64). For comparison, Lo et al. (2012) reviewed the reliability of similar lab-based tasks and reported reliabilities ranging from .56 to .60. The risk-taking task had moderate reliability in all conditions when scores were calculated based on separate modeling (ICC1s ranging from .52 to .65), with reliability increasing to moderate to good when scores were calculated based on joint modeling (ICC1s ranging from .73 to .80). For comparison, in a similar lab-based task, Petzold et al. (2019) report retest correlations between .02 and .65. Finally, the information sampling task had good reliability when scores were calculated based on separate modeling (ICC1 = .78), which further improved when scores were calculated based on joint modeling (ICC1 = .91). For comparison, Grummit et al. (as cited in Enkavi et al., 2019) reported an ICC of .53 in a sample of 312 children and Enkavi et al. (2019) report an ICC of .31 in a computer-based online information sampling task.

**Table 1:** Test–retest reliabilities (ICCs) for the different task measures, analysis approaches, and at 3-week follow-up. Note that for the joint modeling, only ICC1s can be calculated. Additional ICCs (ICC2s) are reported in Supplementary Table S3

Task measure	Separate modeling (ICC1)	Joint modeling (ICC1)	Joint modeling (ICC1, 3 weeks)
Response inhibition task			
Stop signal reaction times	.51	.70	.60
Working memory task			
No distractor (long)	.36	.64	.63
No distractor (short)	.42	.59	.56
Encoding distractor	.34	.51	.51
Delayed distractor	.43	.63	.63
Risk-taking task			
Win	.65	.80	.70
Loss	.57	.73	.67
Mixed	.52	.75	.61
Information sampling task			
Sampling bias	.78	.91	.84

To assess whether reliability decreases with increasing retest periods, we also assessed reliabilities between the first measurement and a 3-week follow-up measurement. As expected, reliabilities for all tasks were slightly lower for the longer retest period (see Table 1).

### Construct validity

To assess construct validity, we used exploratory factor analysis, an approach commonly used to assess discriminant and convergent validity (Eisenberg et al., 2019; Russel, 2002). Factor analysis seeks to reduce the dimensionality of measurements with the aim of revealing common factors underlying several measurements outcomes (Eisenberg et al., 2019). This allows researchers to assess whether measures designed to assess different processes, e.g., cognitive control and decision-making, also capture these differences in the variance of a given dataset. Generally, little is known about the factor structure of smartphone-based experimental task measures. Importantly, the factor structure of measurements can differ depending on the population (Knekta et al., 2019) such as healthy individuals or clinical populations—in our case individuals suffering from SUD. A common concern is that, in the face of known widespread cognitive deficits (Hildebrandt et al., 2021), distinct task measures may load on a single factor, potentially hampering inference on different cognitive processes in patient populations. This concern can be amplified for experiments in the field that utilize a smartphone (as increased external distractions could lead to correlated errors).

**Fig. 3:**
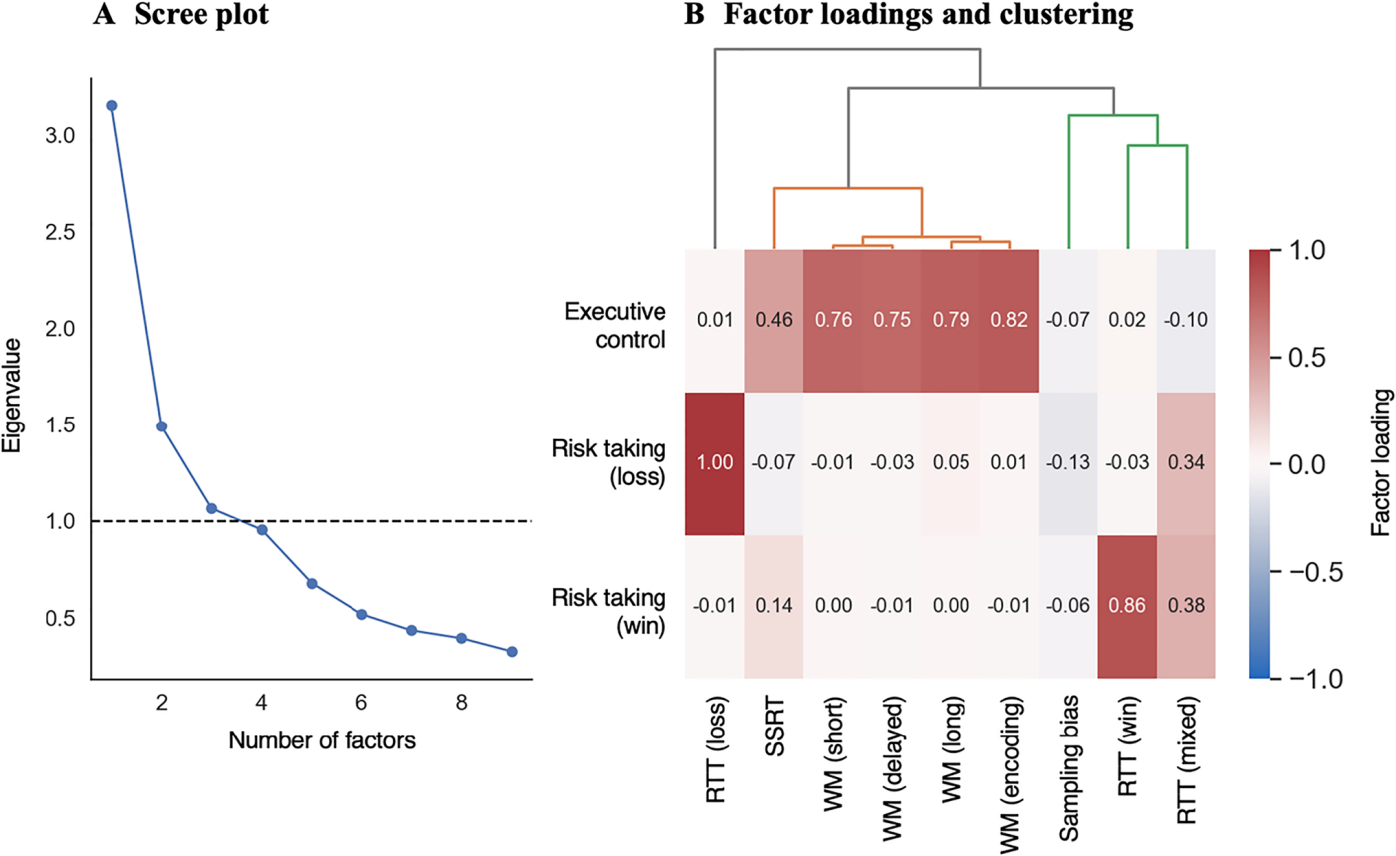
**A** Scree plot used to determine the number of factors best representing the data. **B** Factor loadings for each of the three extracted factors and each of the tasks’ main outcome variables (for explanations of the task outcomes, see Fig. 2). Factor loadings can range from – 1 to 1, where 1 indicates that a variable is fully described by a factor, 0 that there is no relationship between the factor and the variable, and -1 indicates that the variable is fully described by the inverse of the factor. On top of the factor loadings, Panel **B** shows the hierarchical tree diagram generated by the clustering analysis.

We conducted the factor analysis based on the average scores across both sessions from joint modeling, based on evidence that joint modeling yielded the highest reliability estimates. A scree plot indicated that the data was best represented by three factors (see Fig. 3A). Factor loadings indicated the first factor, which we labeled cognitive control (following terminology by, Nigg, 2017), represented measures of working memory and response inhibition. The different conditions in the working memory task had factor loadings of .75 to .82, and the response inhibition task had a factor loading of .46. The second factor represented risk-taking to avoid losses (factor loading of 1.00) and the third factor risk-taking for gains (factor loading of .86). Risk taking for mixed gambles loaded equally on Factor 2 (factor loading of .34) and Factor 3 (factor loading of .38). Information sampling loaded on none of the factors (all loadings ≤ -.06; see Fig. 3). However, an eigenvalue around 1 (Fig. 3A) may indicate, albeit weakly, a separate factor for information sampling (see Supplementary Materials).

Together, the factor loadings indicated a single cognitive control dimension and several decision-making dimensions. However, some measures, such as risk-taking for mixed gambles, did not clearly load on a distinct factor but instead spread over several factors, potentially because they involve a consideration of potential gains and losses. To gain further insight into the structure of the data, we, therefore, conducted a hierarchical clustering analysis based on factor loadings. Rather than showing to which extent a measurement is represented by each individual factor, cluster analyses can reveal which variables load similarly to one or several factors. To illustrate, factors can be understood as dimensions in a “psychological space” and cluster analysis determines how distant task measures are from each other within this space (Eisenberg et al., 2019). Notably, the clusters that emerged from this analysis mirrored the factor analysis regarding a large cognitive control cluster consisting of the working memory and inhibition task (see Fig. 3B). In addition, the analysis revealed two to three decision-making clusters (depending on where one chooses to cut the tree diagram) consisting of one cluster for risk-taking in the context of losses, and one for risk taking when rewards were available. The latter further split into a risk taking for gains and mixed gambles cluster and an information sampling bias cluster. The factor structure, as well as the clustering, are broadly consistent with theoretical predictions of a cognitive control dimension separate from a decision-making dimension that segregates gain and loss contexts (Deza Araujo et al., 2018).

### Reliability of factor scores

Integrating several noisy measures onto latent variables can further increase reliability potentially by reducing measurement error (Eisenberg et al., 2019; Nigg 2017; Shahar et al., 2019). To test whether this was the case in our dataset, we calculated factor scores for each participant and session based on each task measure’s factor loading. The resulting cognitive control scores (based on the cognitive control factor) showed higher test–retest reliability than each of the underlying task measures (ICC1 = .81; compare Table 1). In the decision-making domain, reliabilities were similar to those of the underlying scores both for the risk taking for gains factor (ICC1 = .79) and for the risk taking to avoid losses factor (ICC1 = .82).

### Correlation of task measures with drinking

As proof of concept, we correlated latent factor scores derived from joint modeling with measures of drinking. We observed a correlation between cognitive control and the number of reported drinking days over the last three month (Spearman correlation with Bonferroni corrected *p* values: *r* = – 0.230, 95% CI [– 0.31, – 0.14], *p* < .001, see supplement). When estimating this correlation based on single components of the cognitive control factor derived from separate modeling (focusing on the first session), the five correlations were overall weaker (working memory [short]: *r* = – 0.181, 95% CI [– 0.27, – 0.09]; *p* < .001; working memory [long]: *r* = – 0.131, 95% CI [– 0.22, – 0.04]; *p* = .005; working memory [encoding]: *r* = – 0.187, 95% CI [– 0.27, – 0.10]; *p* < .001; working memory [delayed]: *r* = – 0.210, 95% CI [– 0.30, – 0.12]; *p* < .001; SSRT: *r* = 0.111, 95% CI [– 0.10, 0.08]; *p* = .016). This pattern indicates that improving reliability, through joint modeling and deriving latent factor scores, can uncover significant correlations between task measures and real-world outcomes such as drinking.

## Discussion

We assessed the reliability and validity of four smartphone-based experiments in a large sample of participants suffering from alcohol use disorder. We showed that split-half reliability was high. Test–retest reliability ranged from moderate to good when modeling task data separately for each session, and increased to good to excellent when modeling sessions jointly. This emphasizes a need for adequate modeling of within-subject longitudinal data for sufficient reliability estimates and highlights the value of smartphones for larger data collection than can be accomplished within a laboratory setting. With respect to construct validity, we identified a cognitive control factor distinct from two factors reflecting decision-making in the context of losses versus rewards. Latent variables based on factor loadings further increased test–retest reliability, demonstrating that combining several measures into latent variables is a further useful denoising step. As proof of concept, we demonstrate that a latent cognitive control score based on joint modeling indeed yielded stronger correlations with drinking behavior than single task scores based on separate modeling. Together, our data show that distinct cognitive-motivational aspects can be measured experimentally with sufficient reliability and validity in substance use disorder (SUD) through smartphone-based data collection.

### Improving task reliability through joint modeling

Recent analyses of multiple task measures indicate that most tasks show poor test–retest reliability (Enkavi et al., 2019; Hedge et al. 2018). This poses a major problem because reliability limits the observable correlation between two variables, one possible account of why many task measures show only weak or no correlation to real-life outcomes (Eisenberg et al., 2019). Here, we demonstrate that modeling task outcomes jointly based on two measurement sessions improves a tasks’ test–retest reliability from moderate to good and often excellent levels compared to traditional methods that model each session separately. Previous work indicated that this method of jointly modeling task data produces more reliable laboratory task measures (Brown et al., 2020; Waltmann et al., 2022). A recent simulation showed that this moves reliability estimates closer to true reliability levels by regulating task outcomes based on all available data, thereby reducing measurement noise (Waltmann et al., 2022). Our smartphone tasks are very short (~5 min per task) and the observed improvement rests on data from more than one session and more than one person. Here, smartphone-based tasks have a major advantage over laboratory-based tasks by allowing researchers to collect data more efficiently from several shorter sessions in the field (Miller, 2012; Zech et al., 2020, 2022). Future research could aim at moving tasks to smartphone to obtain reliable scores from two or potentially even more sessions.

### Reliability based on factor scores

In line with prior research (Enkavi et al., 2019; Eisenberg et al., 2019; Shahar et al., 2019), we show that a tasks’ reliability is further increased when measurements are based on factor scores rather than on individual tasks scores. In addition to reducing dimensionality, factor analysis can be regarded as a “denoising” step when data from several related tasks is available. However, having multiple tasks available in the same subjects is relatively unusual and here again smartphone-based tasks can be useful. As participants need to complete more tasks, the burden of participating in experimental sessions in the lab increases (e.g., Eisenberg’s participants had to complete 150 tasks) while smartphone-based tasks can spread this burden over time, thus making participation more engaging for participants.

### State-dependent process?

A major goal of most smartphone-based studies is to detect state-dependent changes. Our data reveal excellent split-half reliability, a finding that is especially encouraging given participants completed the tasks outside a controlled lab environment, where more measurement noise (e.g., because of distractions) could have led to reduced reliability. Moreover, estimates of split-half reliability were consistently higher as compared to test–retest reliability. This indicates that at least part of the unexplained variance in experimental task measures might be driven by state-dependent changes in cognitive and motivational processes (Hedge et al., 2018). As most tasks measuring such processes use cross-sectional designs, little is Zech et al. (2022) known about whether and at what frequency cognitive and motivational processes fluctuate (). There are a few notable exceptions. For example, in a week-long smartphone experiment, Eldar et al. (2018) showed that reward-learning processes fluctuate at two distinct timescales—one fast and one slow—and that these fluctuations were linked to changes in mood. In SUD, Konova et al. (2020) linked longitudinal fluctuations in risky decision making to prospective opioid use. Importantly, they varied the sampling rate (between 1 week and 1 month) and only found the expected association at the weekly sampling rate. In a citizen science sample, smartphone-based assessments of risk-taking were linked to circadian rhythm (Bedder et al., 2020). These studies underline the importance of understanding whether, and at which frequencies, cognitive processes fluctuate. Future research should investigate such fluctuations more deeply by deploying experimental tasks in longitudinal settings. We propose that smartphone-based tasks with sufficient psychometrics, as outlined in the current study, can help in this endeavor. Indeed, they can easily be deployed in real-world environments with high measurement frequencies, and we are currently implementing this in a multi-center study (Heinz et al., 2020).

### Construct validity

Next to reliability, construct validity is an important psychometric criterion. Construct validity refers to the extent to which associations between measurements reflect theoretical relationships between underlying constructs. Although it is as important as reliability, construct validity is rarely assessed for experimental tasks (for an exception, see Eisenberg et al., 2019). We assessed construct validity by creating a psychological space using factor analysis and then categorizing tasks within this psychological space using cluster analysis (following the method described in Eisenberg et al., 2019). This revealed a large cognitive control cluster consisting of working memory and inhibition, in addition to several smaller clusters related to risk taking, the latter broadly splitting into risk taking for gains and risk taking to avoid losses. In line with Eisenberg et al. (2019), our findings do not support the notion of a unifying construct of self-regulation, which may lack coherence as a construct. Instead, we show key factors of cognitive control and motivation that align well with theoretical accounts. In a recent historical review, Nigg (2017) concluded that most theories include working memory and inhibition in cognitive control (see also Botvinik & Braver, 2015), but separate them from decision-making such as risk taking. In a review of behavioral and genetic studies, Friedman and Miyake (2017) further concluded that response inhibition and working memory are robustly correlated. Furthermore, empirical evidence shows a distinction in neural circuits underlying risk taking to avoid losses and risk taking for gains (Deza Araujo et al., 2018). Risk taking for gains decreases with age, putatively reflecting decreases in dopamine (Rutledge et al., 2016), and risk taking for losses has been linked to circadian rhythms (Bedder et al., 2020). This distinction is also in line with prospect theory which posits that people assess risks differently in the context of gains compared to losses (Kahneman & Tversky, 1979; Tversky & Kahneman, 1981). Prospect theory has been extensively tested in both healthy populations and populations with SUD (Cabedo-Peris et al., 2022). We conclude that our tasks have theoretically plausible construct validity. This is the first demonstration of theoretically plausible construct validity for smartphone-based tasks, in this case within a sample of participants suffering from SUD.

### Clinical sample and correlation with drinking

A task’s reliability and construct validity do not only depend on the task itself, but also on other factors such as the investigated sample (Knekta et al., 2019). Most large-scale assessments of task reliability and construct validity have been conducted so far in healthy individuals or rather unselected samples (Eisenberg et al., 2019; Enkavi et al., 2019; Hedge et al. 2018; Shahar et al., 2019; Waltmann et al., 2022). We demonstrate that latent cognitive control factor scores derived from joint modeling yielded higher correlations with a measure of drinking behavior as compared to individual scores derived from separate modeling. This result confirms Spearman’s (1904/2010) prediction that better reliability will increase observed correlations between variables while underlining the importance of increasing reliability through joint longitudinal data modeling and by combining task measures into latent factor scores in clinical populations.

### Limitations

We show that—when analyzing data using traditional analytic approaches—the four tasks tested in this study had already moderate to good reliability, thereby sometimes already exceeding the median reliability of other tasks reported in previous studies (Enkavi et al., 2019; Hedge et al., 2018). The gamification of the tasks may play a role in this regard. For example, gamification of tasks may render tasks more engaging, thereby motivating participants to complete them to the best of their abilities leading to higher between-participant variance and in turn higher reliability. However, our study design did not allow us to systematically assess differences in this regard.

We further demonstrate that one of the extracted factor scores (cognitive control) correlated with real-life measures of drinking in the expected direction (lower cognitive control was related to more drinking). Although this finding is promising with regard to the task measures’ ecological validity, we did not find similar correlations between the two risk taking factors and drinking. On the one hand this finding might point toward a mechanistic insight that cognitive control is more important than risk preferences in SUDs. However, we are hesitant to interpret null-results as it is also possible that characteristics of the task explain these null-results. For example, it is possible that risk taking using abstract rewards does not generalize to risk taking involving real, health-related rewards. Future research could investigate this idea by designing risk taking tasks that are more closely related to risk taking in SUD. It is also possible that increasing the number of trials (for example by including additional measurement sessions) reveals correlations. Finally, future research could explore whether different tasks correlate with other aspects of addictive behaviors that were not investigated in the current project.

The temporal spacing between test sessions is an interesting target for ongoing and future research. Varying this interval systematically could lead to new insights as to when tasks are most sensitive to state- or training-related changes. A noteworthy advantage of our study is that we included two sessions for each task on the first study day, enabling us to specifically assess reliability and validity. Many existing studies on task reliability commonly use data from longitudinal studies that were designed to test clinical or developmental effects (e.g., Shahar et al., 2019, Brown et al., 2020). We also demonstrated that modeling session scores jointly—that is using data from all sessions—further improves reliability to good-to-excellent levels. It should be noted that this approach only works if data from several sessions is available, which might not be feasible for complex studies (e.g., studies involving brain scans). The method is therefore especially advantageous for remote studies such as this one.

One possible limitation of smartphone-based research is, however, that missing data might not be random. For example, it is possible that participants that have generally lower working memory also perform fewer working memory tasks. If data is missing in a systematic way, it should decrease overall variance and make subsequent analyses more conservative. Finally, as both reliability and construct validity depend both on tasks and on populations (Knekta et al., 2019), it should be noted that our results do not necessarily generalize to other populations (e.g., healthy participants or participants with other pathologies).

### Conclusions

We demonstrate good reliability and plausible construct validity of four smartphone-based tasks deployed in a large sample of participants suffering from SUD. We show that reliability can be further improved to good to excellent levels when modeling data from several sessions jointly and when combining several measures into latent variables. Finally, we demonstrate that latent factor scores from joint modeling increase correlations with a measure of drinking. Together, our results demonstrate the strong potential of smartphones to help overcome psychometric shortcomings of lab-based experimental tasks and to investigate real-life outcomes, which require sufficient psychometrics and an easy mobile deployment in real life and in clinically relevant populations. These results represent a critical milestone towards longitudinal experimental studies in SUD research and in psychiatry and psychology more generally.

## Materials and methods

### General procedure

This study was part of a larger German research consortium on substance use disorder (SUD) at three sites (Technical University Dresden, Charité Berlin, and Central Institute of Health Mannheim), in which a smartphone-based longitudinal Ecological Momentary Assessment (EMA) of up to 1 year was performed with a range of subjective reports. In addition to subjective reports, individuals performed four cognitive-motivational tasks on the smartphone once per month. These tasks were taken from the Great Brain Experiment (GBE) app (Brown et al., 2014, see below for details). Before starting the EMA study, individuals underwent extensive clinical and neurocognitive assessments (see Heinz et al., 2020). During this assessment appointment, which was either conducted inside the laboratory or online via video chat, the app for running the EMA study (Movisens app; movisens GmbH, Germany; Reichert et al., 2021) as well as a customized version of the GBE app for assessment of the four cognitive-motivational tasks (see below) were installed either on participants’ own phone or on a study phone. On the first Monday following the assessment, participants were prompted to complete each smartphone task twice. The current study focusses on these first two measurement sessions. Participants also participated in multiple sub-projects of the consortium (see Heinz et al., 2020), that are unrelated to the present study.

### Participants

The study procedure was approved by the review boards of the local ethics committee at Heidelberg University (2018-621N-MA), Charité – Universitätsmedizin Berlin (EA1/212/18), and Technical University Dresden (EK 459112018). Data collection took place between February 2020 and March 2022. All participants gave written informed consent before participating in the study. For study inclusion at all three sites, participants had to fulfill criteria of substance used disorder, specifically mild to moderate Alcohol Use Disorder (AUD). According to DSM 5, mild to moderate AUD was defined as the presence of at least two AUD criteria. Participants were recruited through flyers and advertisements. Telephone screenings were conducted before study inclusion/exclusion. Exclusion criteria were: clinical indication for detoxification treatment, insufficient knowledge of the German language, seeking a therapeutic intervention, MRI contraindications, medical history of DSM-5 bipolar disorder, psychotic disorder, schizophrenia or schizophrenic spectrum disorder, or current use of drugs or medication nor substance dependence thereof other than alcohol, nicotine, or cannabis, as well as medical history of severe head injury, or other severe central nervous system disorders. Data from 488 participants was analyzed for the present study. Participants age ranged from 16 to 65 years (*M* = 36.9, *SD* = 12.8) and 180 participants (36.9%) reported to be female. Participants fulfilled 2 to 9 AUD criteria ranged from (*M* = 4.05, *SD* = 1.60).

### Inhibition task

During the Inhibition Task (Smittenaar et al., 2015), participants tapped left or right on their smartphone screen to collect fruits falling from a tree (see Fig. 1). Each trial began with two fruits hanging at the top of the screen for one to three seconds (randomly selected from a uniform distribution). Next, one of the fruits fell down and passed over one of two circles indicating the time during which participants should collect the fruit through tapping (Go-Trials with a response window spanning from 500 to 800 ms after stimulus onset). In 12 of 32 trials (37.5%), the falling fruit turned brown, indicating that it was rotten and should not be collected (stop trials). At the beginning of each session the delay after which the fruit turned brown (stop signal delay; SSD) was 350 ms. This delay changed according to staircase procedure (Verbruggen et al., 2019): it increased by 50 ms after each successful stop trial (rendering the subsequent stop trial more difficult) and decreased by 50 ms after each unsuccessful stop trial (rendering the subsequent stop trial easier).

### Working memory task

During the working memory task (McNab et al., 2015), participants were asked to remember the positions of two up to 12 red circles presented on a 4 x 4 grid (see Fig. 1). The task involved four conditions: In the ‘*long no distractor’* condition circles were presented for 2 s (encoding phase), then disappeared for 1 s (maintenance phase), before participants had to tap on their no-longer visible locations. In the ‘short *no-distractor’* condition, patterns were presented for 1 instead of 2 s. In the ‘*encoding-distractor’* condition, two yellow distractors were presented together with the red circles during the encoding phase. In the ‘*delayed-distractor’* condition, the same two yellow distractors were presented but during the maintenance phase. Each condition started with three circles in trial one. If participants failed to respond correctly, two circles were presented in the second trial. If participants failed at this level, the condition was terminated. If a trial was completed correctly, the number of red circles in the corresponding condition increased by one in the next trial. If participants failed in a trial (from level four onwards), the level was repeated once. If they failed again the condition was terminated. A maximum of eight trials was completed for each condition.

### Risk taking task

During the risk taking task (Rutledge et al., 2014), participants repeatedly chose between a certain outcome and a gamble, with equal probabilities of the two outcomes (see Fig. 1). The task involved three conditions: In the ‘*gain’* condition participants chose between either a certain gain or to gamble for a larger gain against 0 points. In the ‘*loss’* condition, participants chose between either a certain loss or to gamble for 0 points against a larger loss. In the ‘*mixed’* condition, participants chose between a certain amount of 0 points or to gamble for a gain against a loss amount. The gain and loss conditions consisted of 11 trials and the mixed condition consisted of eight trials. In each trial, a certain amount was first randomly chosen with replacement from a fixed list of outcomes. Gamble amounts were then calculated by multiplying the certain amount with a randomly chosen multiplier from another fixed list (for details, see Bedder et al., 2020; Rutledge et al., 2014). The task also involved current mood ratings (“How happy are you at this moment?”; rating line with endpoints “very happy” and “very unhappy”) which were presented after every 2–3 trials and are known to be correlated with depressive symptoms (Rutledge et al., 2014), but are not subject to the currently reported reliability analysis.

### Information sampling task

During the information sampling task (Hunt et al., 2016) participants were presented with four playing cards in rows of two and had to choose the row with the largest sum of card values (see Fig 1). Each of the 21 trials began with all cards facing down. Participants could invest points to turn over one card at a time to sample information with increasing costs for each additional card (zero points for the first card, 10 for the first card, 15 for the third, and 20 for the fourth card). Before turning over a card, participants could also choose to guess, at no cost, which row had the largest value. A choice at this stage would be a gamble (called a guess in the task) at 50/50. Participants won 60 points if this guess was correct and lost 50 points if the guess was incorrect. If turning over one or multiple cards, the costs for information sampling reduced the total win. Card values were sampled randomly with replacement from a discrete uniform distribution with integers ranging from 1–10.

### Reliability

The first goal of this study was to assess the smartphone tasks’ reliability. Where possible, we first assessed the tasks’ split-half reliability, i.e., the consistency with which a task measures its construct within one measurement session. Next, we assessed the tasks’ test–retest reliability, i.e., the consistency with which a task measures its construct between two measurement sessions. While assessing the tasks’ test–retest reliability, we compared two approaches of analyzing task data—the more traditional approach in which sessions are modeled separately, and an alternative approach in which sessions are modeled jointly. The latter has recently been shown to yield superior reliability estimates in theory and practice in other cognitive tasks (for details, see below; Brown, 2020; Haines et al., 2020; Waltmann et al., 2022).

### Modeling sessions separately vs. jointly

For each task, we compared two approaches of analyzing task data: The first approach, which we subsequently call *separate modeling*, is traditionally used to analyze task data. In this approach, summary scores are first created by aggregating data separately for each session of each participant. Next, these summary scores are used for inference, for example to calculate test–retest reliabilities. According to Haines et al. (2020), one problem of this approach is that it assumes that scores are estimated without measurement error. This, in turn, leads to ignoring uncertainty during inference, which, for example, can attenuate test–retest reliability. A second problem is that this method assumes that person-level parameters are distributed uniformly across an interval that spans beyond a reasonable range of task scores. This is because knowledge about scores from other participants or scores from other sessions of the same participant is not integrated in estimating individual session scores. Prior research shows that integrating such information into individual score estimation yields more reliable scores (Brown et al., 2020; Efron & Morris, 1977; Haines et al., 2020; Rouder & Haaf, 2019; Waltmann et al., 2022; Williams et al., 2021).

The alternative analysis approach, which we subsequently call *joint modeling*, overcomes both problems of the separate modeling approach. Instead of first calculating summary scores and using them in a second step for inference, the prediction approach performs inference directly based on all available trial-level data. This allows it to carry, firstly, within-session uncertainty into the inference step and, secondly, to use information from other participants and sessions in each individual session score estimation. Both of these aspects improved test–retest reliability in previous work. We implemented this approach using hierarchical mixed models specifically designed to model each task’s outcome measure (for details see Supplementary Materials). Hierarchical mixed models allow us to analyze data at the trial-level while still accounting for the participant and session structure of the data. We validated that scores based on mixed models did not substantially differ from task scores calculated with established methods when modelled for each session separately (see Supplementary Materials).

### Split-half and test–retest reliability

Firstly, split-half reliability was assessed based on Spearman–Brown-corrected correlations within each session (based on odd-even splits). Note that for the working memory task and for the inhibition task, split-half reliabilities could not be computed because these tasks are adaptive. Therefore, splitting the task into two halves is not appropriate (Draheim et al., 2020). Qualitative interpretations of split-half reliabilities are given in line with Nunnally and Bernstein (1994; split-half reliabilities above .8 were labeled as adequate). Secondly, test–retest reliability was calculated based on intra-class correlation coefficients (ICCs) based on data from the first two measurement sessions. To calculate ICCs directly from mixed models, we followed the method recently described by Brown et al. (2020), which calculates reliabilities based on variance components extracted from mixed models. Waltmann et al. (2022) recently showed that this method yields more conservative and more accurate reliabilities than alternative methods (e.g., first predicting sessions scores and calculating reliabilities based on these predictions) and thus crucially avoids overestimating reliability. Qualitative interpretations of test–retest reliabilities are given in accordance with Koo and Li (2016): ICCs less than .5 were being interpreted as “poor”, ICCs between .5 and .75 as “moderate”, ICCs between .75 and .9 as “good”, and ICCs above .9 as “excellent”.

### Factor and clustering analysis

Exploratory factor analysis was conducted using maximum likelihood estimation followed by oblimin rotation, which rotates factors without enforcing orthogonality. The analysis was based on average joint prediction scores from both sessions. Before conducting this analysis, the outcome measure of the inhibition task was inverted, so that it could be interpreted in the same direction as the outcome measure of the working memory task (i.e., higher values indicating better performance). This analysis was implemented using the factor_analyzer package (Python 3.5). The optimal number of factors was determined using a scree plot (see Fig. 3). The hierarchical clustering analysis was conducted using the SciPy package (Python 3.5). The analysis was conducted using Euclidean distances to generate a hierarchical tree. As there were no implicit heights at which to cut this tree, the cut height was determined based on theoretical considerations. To calculate reliability of factor scores, factor scores were extracted separately for each session using the tenBerge method, which is most appropriate for oblimin rotation (Ten Berge et al., 1999).

### Supplementary Information

Below is the link to the electronic supplementary material.

Supplementary file1 (DOCX 522 KB)

## Footnotes

1 Note that all comparison studies used a traditional, separate modeling approach.

## References

[CR1] Bedder, R., Vaghi, M., Dolan, R., & Rutledge, R. (2020). Risk taking for potential losses but not gains increases with time of day. *PsyArXiv.*10.31234/osf.io/3qdnx 10.1038/s41598-023-31738-x PMC10073197 37015952

[CR2] Berkman ET, Falk EB, Lieberman MD (2011). In the trenches of real-world self-control. Psychological Science.

[CR3] Botvinick M, Braver T (2015). Motivation and cognitive control: From behavior to neural mechanism. Annual Review of Psychology.

[CR4] Brown HR, Zeidman P, Smittenaar P, Adams RA, McNab F, Rutledge RB, Dolan RJ (2014). Crowdsourcing for cognitive science–the utility of smartphones. PLoS One.

[CR5] Brown VM, Chen J, Gillan CM, Price RB (2020). Improving the reliability of computational analyses: Model-based planning and its relationship with compulsivity. Biological Psychiatry: Cognitive Neuroscience and Neuroimaging.

[CR6] Cabedo-Peris, J., González-Sala, F., Merino-Soto, C., Pablo, J. Á. C., & Toledano-Toledano, F. (2022). Decision making in addictive behaviors based on prospect theory: A systematic review. *Healthcare, 10*(9), 1659. 10.3390/healthcare10091659 PMC9498454 36141271

[CR7] Deza Araujo YI, Nebe S, Neukam PT, Pooseh S, Sebold M, Garbusow M, Smolka MN (2018). Risk seeking for losses modulates the functional connectivity of the default mode and left frontoparietal networks in young males. Cognitive, Affective, & Behavioral Neuroscience.

[CR8] Draheim, C., Tsukahara, J. S., Martin, J. D., Mashburn, C. A., & Engle, R. W. (2020). A toolbox approach to improving the measurement of attention control. *Journal of Experimental Psychology: General. Advance online publication.* 10.1037/xge0000783 32700925

[CR9] Efron B, Morris C (1977). Stein's paradox in statistics. Scientific American.

[CR10] Eisenberg IW, Bissett PG, Zeynep Enkavi A, Li J, MacKinnon DP, Marsch LA, Poldrack RA (2019). Uncovering the structure of self-regulation through data-driven ontology discovery. Nature Communications.

[CR11] Ekhtiari H, Victor TA, Paulus MP (2017). Aberrant decision-making and drug addiction—How strong is the evidence?. Current Opinion in Behavioral Sciences.

[CR12] Eldar E, Roth C, Dayan P, Dolan RJ (2018). Decodability of reward learning signals predicts mood fluctuations. Current Biology.

[CR13] Enkavi AZ, Eisenberg IW, Bissett PG, Mazza GL, MacKinnon DP, Marsch LA, Poldrack RA (2019). Large-scale analysis of test–retest reliabilities of self-regulation measures. Proceedings of the National Academy of Sciences.

[CR14] Falk A, Heckman JJ (2009). Lab experiments are a major source of knowledge in the social sciences. Science.

[CR15] Friedman, N. P., & Miyake, A. (2017). Unity and diversity of executive functions: Individual differences as a window on cognitive structure. *Cortex,**86*, 186–204. 10.1016/j.cortex.2016.04.023 10.1016/j.cortex.2016.04.023 PMC5104682 27251123

[CR16] Goschke T (2014). Dysfunctions of decision-making and cognitive control as transdiagnostic mechanisms of mental disorders: Advances, gaps, and needs in current research. International Journal of Methods in Psychiatric Research.

[CR17] Haines, N., Kvam, P. D., Irving, L. H., Smith, C., Beauchaine, T. P., Pitt, M. A., ... & Turner, B. M. (2020). Theoretically informed generative models can advance the psychological and brain sciences: Lessons from the reliability paradox. 10.1037/met0000674 40232753

[CR18] Hedge C, Powell G, Sumner P (2018). The reliability paradox: Why robust cognitive tasks do not produce reliable individual differences. Behavior Research Methods.

[CR19] Heinz A, Kiefer F, Smolka MN, Endrass T, Beste C, Beck A, Spanagel R (2020). Addiction research consortium: Losing and regaining control over drug intake (ReCoDe)—From trajectories to mechanisms and interventions. Addiction Biology.

[CR20] Hildebrandt MK, Dieterich R, Endrass T (2021). Neural correlates of inhibitory control in relation to the degree of substance use and substance-related problems–a systematic review and perspective. Neuroscience & Biobehavioral Reviews.

[CR21] Hunt LT, Rutledge RB, Malalasekera WN, Kennerley SW, Dolan RJ (2016). Approach-induced biases in human information sampling. PLoS Biology.

[CR22] Kahneman Daniel, Tversky Amos (1979). Prospect Theory: An Analysis of Decision under Risk. Econometrica.

[CR23] Knekta E, Runyon C, Eddy S (2019). One size doesn’t fit all: Using factor analysis to gather validity evidence when using surveys in your research. CBE—Life $ciences Education.

[CR24] Konova AB, Lopez-Guzman S, Urmanche A, Ross S, Louie K, Rotrosen J, Glimcher PW (2020). Computational markers of risky decision-making for identification of temporal windows of vulnerability to opioid use in a real-world clinical setting. JAMA Psychiatry.

[CR25] Koo TK, Li MY (2016). A guideline of selecting and reporting intraclass correlation coefficients for reliability research. Journal of Chiropractic Medicine.

[CR26] Kräplin A, Scherbaum S, Bühringer G, Goschke T (2016). Retest reliabilities of decision-making and cognitive control measures in addictive disorders. Sucht.

[CR27] Krönke KM, Mohr H, Wolff M, Kraplin A, Smolka MN, Buhringer G, Ruge H, Goschke T (2021). Real-life self-control is predicted by parietal activity during preference decision making: A brain decoding analysis. Cognitive, Affective, & Behavioral Neuroscience.

[CR28] Krönke KM, Wolff M, Mohr H, Kräplin A, Smolka MN, Bühringer G, Goschke T (2018). Monitor yourself! Deficient error-related brain activity predicts real-life self-control failures. Cognitive, Affective, & Behavioral Neuroscience.

[CR29] Krönke KM, Wolff M, Mohr H, Kräplin A, Smolka MN, Bühringer G, Goschke T (2020). Predicting real-life self-control from brain activity encoding the value of anticipated future outcomes. Psychological Science.

[CR30] Krönke KM, Mohr H, Wolff M, Kraplin A, Smolka MN, Buhringer G, Ruge H, Goschke T (2021). Real-life self-control is predicted by parietal activity during preference decision making: A brain decoding analysis. Cognitive, Affective, & Behavioral Neuroscience.

[CR31] Krönke KM, Wolff M, Shi Y, Kräplin A, Smolka MN, Bühringer G, Goschke T (2020). Functional connectivity in a triple-network saliency model is associated with real-life self-control. Neuropsychologia.

[CR32] Liljequist, D., Elfving, B., & Skavberg Roaldsen, K. (2019). Intraclass correlation–A discussion and demonstration of basic features. *PloS one, 14*(7), e0219854. 10.1371/journal.pone.0219854 10.1371/journal.pone.0219854 PMC6645485 31329615

[CR33] Lo, A. H., Humphreys, M., Byrne, G. J., & Pachana, N. A. (2012). Test–retest reliability and practice effects of the Wechsler Memory Scale‐III. *Journal of Neuropsychology, 6*(2), 212–231. 10.1111/j.1748-6653.2011.02023.x 22257421

[CR34] Lopez RB, Hofmann W, Wagner DD, Kelley WM, Heatherton TF (2014). Neural predictors of giving in to temptation in daily life. Psychological Science.

[CR35] McNab F, Zeidman P, Rutledge RB, Smittenaar P, Brown HR, Adams RA, Dolan RJ (2015). Age-related changes in working memory and the ability to ignore distraction. Proceedings of the National Academy of Sciences.

[CR36] Miller G (2012). The smartphone psychology manifesto. Perspectives on Psychological Science.

[CR37] Miller J, Ulrich R (2013). Mental chronometry and individual differences: Modeling reliabilities and correlations of reaction time means and effect sizes. Psychonomic Bulletin & Review.

[CR38] Moffitt TE, Arseneault L, Belsky D, Dickson N, Hancox RJ, Harrington H, Caspi A (2011). A gradient of childhood self-control predicts health, wealth, and public safety. Proceedings of the National Academy of Sciences.

[CR39] Nigg JT (2017). Annual research review: On the relations among self-regulation, self-control, executive functioning, effortful control, cognitive control, impulsivity, risk-taking, and inhibition for developmental psychopathology. Journal of Child Psychology and Psychiatry.

[CR40] Nunnally J, Bernstein IH (1994). *Psychometric theory*.

[CR41] Overmeyer R, Berghauser J, Dieterich R, Wolff M, Goschke T, Endrass T (2021). The error-related negativity predicts self-control failures in daily life. Frontiers in Human Neuroscience.

[CR42] Petzold J, Kienast A, Lee Y, Pooseh S, London ED, Goschke T, Smolka MN (2019). Baseline impulsivity may moderate L-DOPA effects on value-based decision-making. Scientific Reports.

[CR43] Reichert M, Gan G, Renz M, Braun U, Brüßler S, Timm I, Meyer-Lindenberg A (2021). Ambulatory assessment for precision psychiatry: Foundations, current developments and future avenues. Experimental Neurology.

[CR44] Rouder JN, Haaf JM (2019). A psychometrics of individual differences in experimental tasks. Psychonomic Bulletin & Review.

[CR45] Russell DW (2002). In search of underlying dimensions: The use (and abuse) of factor analysis in personality and social psychology bulletin. Personality and Social Psychology Bulletin.

[CR46] Rutledge RB, Skandali N, Dayan P, Dolan RJ (2014). A computational and neural model of momentary subjective well-being. Proceedings of the National Academy of Sciences.

[CR47] Rutledge RB, Smittenaar P, Zeidman P, Brown HR, Adams RA, Lindenberger U, Dolan RJ (2016). Risk taking for potential reward decreases across the lifespan. Current Biology.

[CR48] Shahar N, Hauser TU, Moutoussis M, Moran R, Keramati M, Consortium N, Dolan RJ (2019). Improving the reliability of model-based decision-making estimates in the two-stage decision task with reaction-times and drift-diffusion modeling. PLoS Computational Biology.

[CR49] Smittenaar P, Rutledge RB, Zeidman P, Adams RA, Brown H, Lewis G, Dolan RJ (2015). Proactive and reactive response inhibition across the lifespan. PLoS One.

[CR50] Spearman, C. (1904/2010). The proof and measurement of association between two things. *International Journal of Epidemiology,**39*, 1137–1150. (Original work published 1904). 10.1093/ije/dyq191 21051364

[CR51] Stavro K, Pelletier J, Potvin S (2013). Widespread and sustained cognitive deficits in alcoholism: A meta-analysis. Addiction Biology.

[CR52] Ten Berge JM, Krijnen WP, Wansbeek T, Shapiro A (1999). Some new results on correlation-preserving factor scores prediction methods. Linear Algebra and its Applications.

[CR53] Tversky, A., & Kahneman, D. (1981). The framing of decisions and the psychology of choice. *Science*, *211*, 453–458. 10.1126/science.7455683 7455683

[CR54] Verbruggen F, Aron AR, Band GP, Beste C, Bissett PG, Brockett AT, Boehler CN (2019). A consensus guide to capturing the ability to inhibit actions and impulsive behaviors in the stop-signal task. eLIFE.

[CR55] Waltmann, M., Schlagenhauf, F., & Deserno, L. (2022). Sufficient reliability of the behavioral and computational readouts of a probabilistic reversal learning task. *Behavior Research Methods*, 1–22. 10.3758/s13428-021-01739-7 PMC9729159 35167111

[CR56] Williams DR, Mulder J, Rouder JN, Rast P (2021). Beneath the surface: Unearthing within-person variability and mean relations with Bayesian mixed models. Psychological Methods.

[CR57] Zech HG, Rotteveel M, van Dijk WW, van Dillen LF (2020). A mobile approach-avoidance task. Behavior Research Methods.

[CR58] Zech, H. G., Reichert, M., Ebner-Priemer, U. W., Tost, H., Rapp, M. A., Heinz, A., et al. (2022). Mobile data collection of cognitive-behavioral tasks in substance use disorders: Where are we now? *Neuropsychobiology*, 1–13. 10.1159/000523697 35350031

