# Supplementary material for: Measuring self-regulation in everyday life: Reliability and validity of smartphone-based experiments in alcohol use disorder
Source: Behav Res Methods. 2022 Dec 12;55(8):4329–42. doi: 10.3758/s13428-022-02019-8 (PMC10700450; doi:10.3758/s13428-022-02019-8)
Supplement: Supplementary file 1 — Supplementary file1 (DOCX 522 KB) [file 13428_2022_2019_MOESM1_ESM.docx]

**Measuring self-regulation in everyday life: reliability and validity of smartphone-based experiments in alcohol use disorder**

**Supplementary materials**

Hilmar Zech* ^1,2^, Maria Waltmann^2,3^, Ying Lee^1,4,5^, Markus Reichert^6,7,8^, Rachel L. Bedder^4,5,9^, Robb B. Rutledge^4,5,10^, Friederike Deeken11, Julia Wenzel12 Friederike Wedemeyer^12^, Alvaro Aguilera13, Acelya Aslan14, Patrick Bach^14^, Nadja S. Bahr^12^, Claudia Ebrahimi^12^, Pascale C. Fischbach^1^, Marvin Ganz^14^; Maria Garbusow^12^, Charlotte M. Großkopf^1^, Marie Heigert^12^, Angela Hentschel^1^, Matthew Belanger^1^; Damian Karl^14^, Patricia Pelz^12^, Mathieu Pinger15, Carlotta Riemerschmid^12^, Annika Rosenthal^12^ Johannes Steffen^1^, Jens Strehle^13^, Franziska Weiss^15^, Gesine Wieder^13^, Alfred Wieland^14^, Judith Zaiser^14^, Sina Zimmermann^14^; Shuyan Liu^12^, Thomas Goschke16, Henrik Walter^12^, Heike Tost^8^, Bernd Lenz^14^, Jamila Andoh^8^, Ulrich Ebner-Priemer^7,8^, Michael A. Rapp^11,^ , Andreas Heinz^12^, Ray Dolan^4,5,^17^,^18, Michael N. Smolka^1^, Lorenz Deserno* ^1,2,^3

and the ReCoDe-Consortium

Department of Psychiatry, Technische Universität Dresden, Dresden, Germany

2 Department of Child and Adolescent Psychiatry, Psychosomatics and Psychotherapy, Centre of Mental Health, University of Würzburg, Margarete-Höppel-Platz 1, 97080 Würzburg, Germany

3 Max Planck Institute for Human Cognitive and Brain Sciences, Stephanstraße 1, 04103 Leipzig, Germany

4 Max Planck University College London Centre for Computational Psychiatry and Ageing Research, London, UK

5 Wellcome Centre for Neuroimaging (WCHN), University College London, London, UK

6 Department of eHealth and Sports Analytics, Faculty of Sport Science, Ruhr-Universität Bochum (RUB), Bochum, Germany

7 Mental mHealth Lab, Institute of Sports and Sports Science, Karlsruhe Institute of Technology (KIT), Karlsruhe, Germany

8 Department of Psychiatry and Psychotherapy, Central Institute of Mental Health, Medical Faculty Mannheim, Heidelberg University, Mannheim, Germany

9 Neuroscience Institute & Department of Psychology, Princeton University, Princeton, NJ, USA

10 Department of Psychology, Yale University, New Haven, CT, USA

11 Social and Preventive Medicine, Department of Sports and Health Sciences, Intra-faculty unit “Cognitive Sciences”, Faculty of Human Science, and Faculty of Health Sciences Brandenburg, Research Area Services Research and e-Health, University of Potsdam, Potsdam, Germany

12 Department of Psychiatry and Neurosciences | CCM, Charité – Universitätsmedizin Berlin, corporate member of Freie Universität Berlin and Humboldt-Universität zu Berlin, Department of Pediatric Surgery, Augustenburger Platz 1, 13353 Berlin, Germany

13 Center for Information Services and High Performance Computing (ZIH), Technische Universität Dresden, Dresden, Germany

14 Department of Addictive Behavior and Addiction Medicine, Central Institute of Mental Health, Medical Faculty Mannheim, Heidelberg University, Mannheim, Germany

15 Department of Clinical Psychology, Central Institute of Mental Health, Medical Faculty Mannheim, University of Heidelberg, Mannheim, Germany

16 Department of Psychology, Technische Universität Dresden, Dresden, Germany

17 State Key Laboratory of Cognitive Neuroscience and Learning, IDG/McGovern Institute for Brain Research, Beijing Normal University, Beijing, China

18 BIH Visiting Professor, Stiftung Charité, Department of Psychiatry and Psychotherapy, Charité – Universitätsmedizin, Berlin, Germany

*Corresponding authors: Hilmar Zech and Lorenz Deserno.

**Email:**  [hilmar.zech@tu-dresden.de](mailto:hilmar.zech@tu-dresden.de) / [deserno_l@ukw.de](mailto:deserno_l@ukw.de)

**Participant characteristics**

**Table S1.** Participant characteristics.

| Participant characteristics (N = 488) | Mean (SD) [range] or percentage |
| --- | --- |
| Age (years) | 36.9 (12.8) [16–65] |
| Women | 180 (36.9%) |
| AUD criteria | 4.05 (1.60) [2–9] |

**Additional ICCs**

**Table S2.** Additional ICCs.

|  | Separate modeling | | Joint modeling |
| --- | --- | --- | --- |
| Task measure | ICC(1) | ICC(2) | ICC(1) |
| **Response inhibition task** |  |  |  |
| SSRT | .52 | .53 | .70 |
| **Working memory task** |  |  |  |
| No distractor (long) | .34 | .34 | .61 |
| No distractor (short) | .42 | .42 | .57 |
| Encoding distractor | .34 | .34 | .51 |
| Delayed distractor | .42 | .43 | .61 |
| **Risk taking task** |  |  |  |
| Win | .65 | .65 | .79 |
| Loss | .54 | .55 | .70 |
| Mixed | .49 | .50 | .72 |
| **Information sampling task** |  |  |  |
| Sampling bias | .78 | .78 | .91 |

**Joint modeling of task data**

In this study, we implemented the joint modeling approach by translating traditional aggregation-based analyses into analyses using hierarchical mixed models. Hierarchical mixed models allow to analyze data at the trial-level while still accounting for the participant and session structure of the data. To verify that this translation did not substantially change task scores, we also included a third analysis approach, in which we used mixed models but modeled data separately for each session. In the following, we will describe for each task how more traditional, aggregation-based scores are computed and how we translated these calculations into trial-level analyses using hierarchical mixed models.

***Stop Signal Task.*** The primary outcome of the stop signal task is the stop signal reaction time (SSRT), which captures the latency in inhibiting the response (Verbruggen et al., 2019). As this latency cannot be observed directly, it is estimated from the go-reaction times (RTs), stop signal delays (SSDs), and stop trial accuracy. Specifically, SSRTs are estimated by first integrating the RT distribution and finding the point at which the integral equals the probability of responding correctly after a stop signal. Next, the mean SSD is subtracted from the result of this calculation to yield the SSRT. This method does not lend itself well to modelling at the trial level. To generate mixed models to estimate SSRTs, we therefore instead used an often implemented simplified method of calculating SSRTs, in which the SSRTs are calculated by subtracting the mean SSD from the mean RT. This model was translated to the following mixed regression models (for notation, see Bates, 2005):

*ssd_rt ~ 1 + (1 | is_stop / participant / session)*

Here *ssd_rt* refers to a variable consisting of stop signal delay times or reaction times (depending on the trial type) and *is_stop* to the trial type (stop or go trial). Predicted session scores from this model correlated highly with scores calculated by the integration method (*r* = .93).

***Working memory task.*** The primary outcome of the working memory task is the maximum level participants reach within each condition. To create equivalent scores in a mixed model, we first imputed missing data from levels that participants did not reach by setting the accuracy for these levels to zero. Next, we predicted trial-level accuracy using the following binomial mixed model:

*accuracy ~ 1 + (1 | participant/session)*

Predicted session scores from these models correlated highly with traditionally calculated maximum level reached scores (*r*s > .99; see Table S3).

***Risk taking task.*** The primary outcome of the risk taking task is the percentage of gambles for each condition. This score can easily be calculated with binomial mixed models that predict gambling at a trial level:

*is_gamble ~ 1 + (1 | participant/session)*

Here *is_*gamble refers to whether a participant gambled or not in a given trial. Predicted session scores from these models correlated highly with traditionally aggregated scores (*r*s > .99; see Table S3).

***Information sampling task.*** The primary outcome of the information sampling task is average information oversampling in each session. Oversampling for each trial is calculated as the difference between turned around cards and the optimal number of card turns, derived from a normative dynamic programming model (Hunt et al., 2016). The dynamic programming model calculates optimal information sampling by calculating the expected value of every possible action (seeking more information vs guessing) for each step of the task (see supplementary materials; Hunt et al., 2016). We calculated model-derived scores by predicting oversampling using a mixed model:

*oversampling ~ 1 + (1 | participant / session)*

The predicted scores from this model correlated highly with traditionally aggregated scores (*r* > .99).

**Table S3.** Correlations between aggregated and modeled scores.

| Task measure | Correlation |
| --- | --- |
| **Response inhibition task** |  |
| SSRT | .93 |
| **Working memory task** |  |
| No distractor (long) | >.99 |
| No distractor (short) | >.99 |
| Encoding distractor | >.99 |
| Delayed distractor | >.99 |
| **Risk taking task** |  |
| Win | >.99 |
| Loss | >.99 |
| Mixed | >.99 |
| **Information sampling task** |  |
| Sampling bias | >.99 |

**Four factor solution**


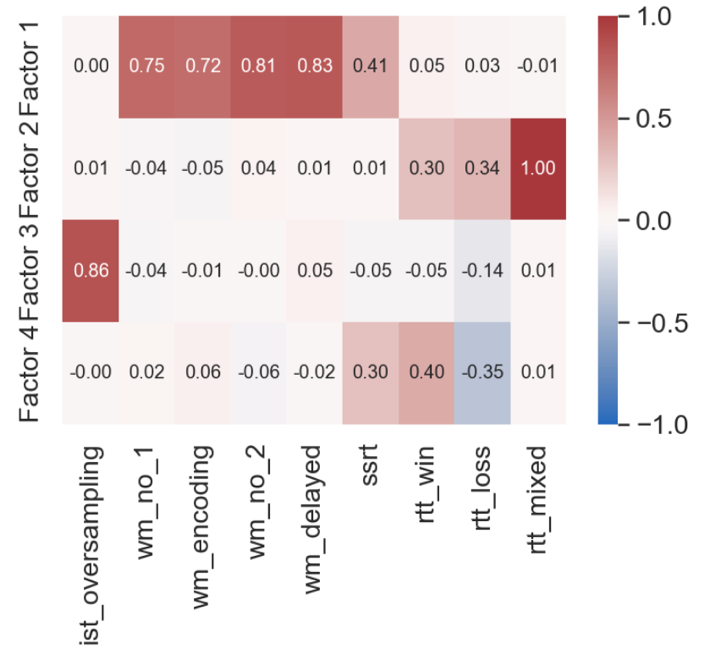


**Figure S4.** This figure shows factor loadings of each task measurement for a four factor solution.

**
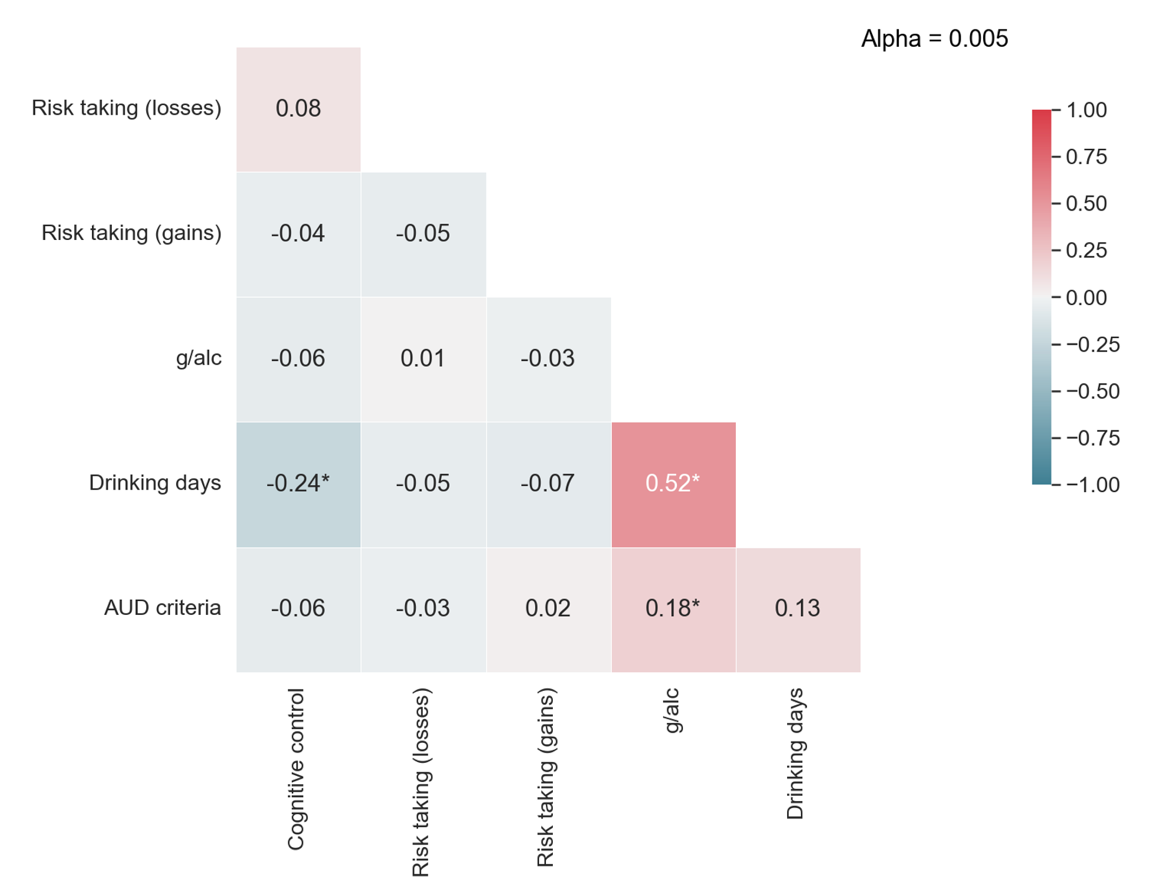
Correlations with drinking behavior**

**Figure S5.** Correlation between factor scores and retrospective drinking.


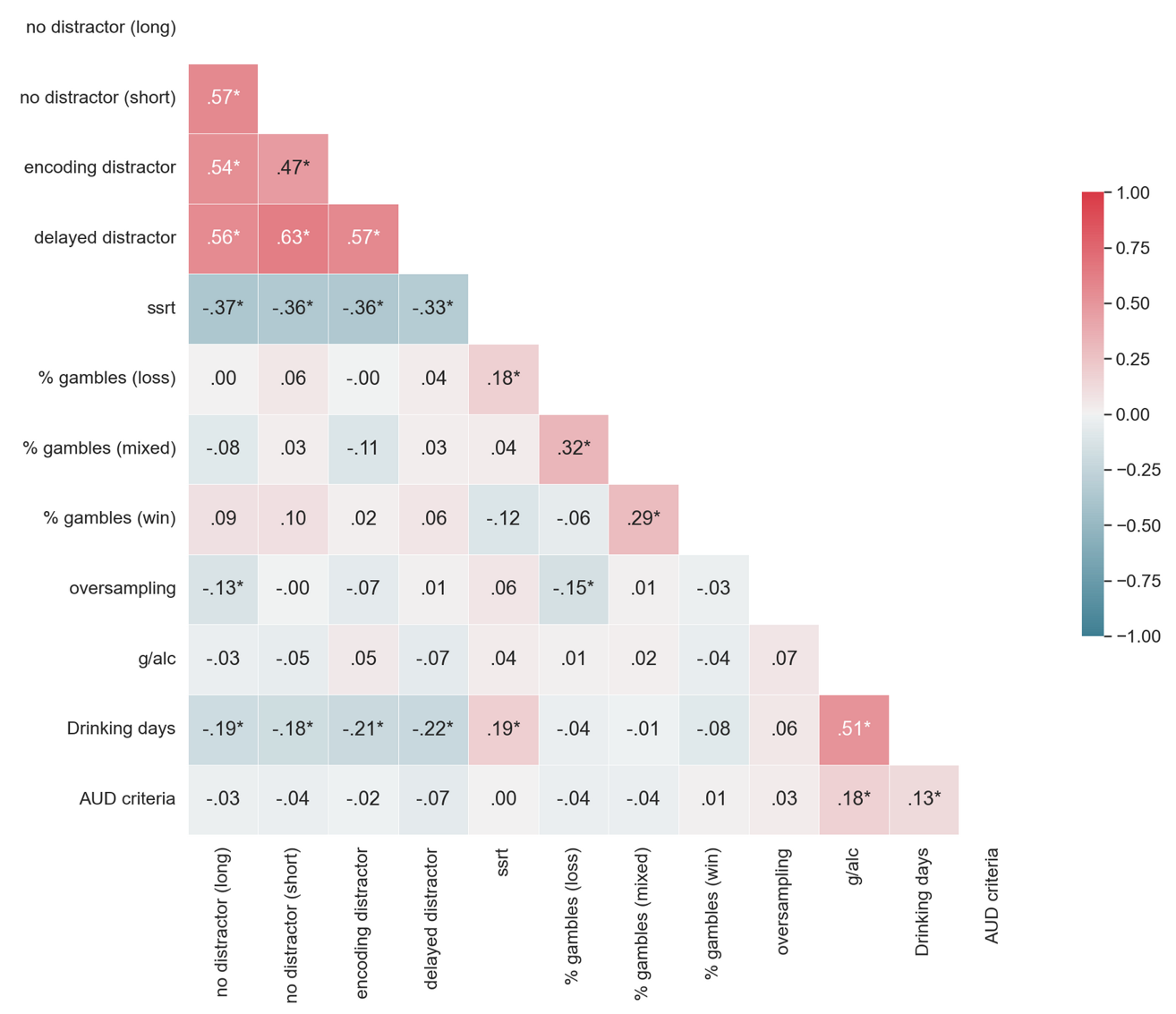


**Figure S6.** Correlation between task scores and retrospective drinking.
